# DepthCrackNet: A Deep Learning Model for Automatic Pavement Crack Detection

**DOI:** 10.3390/jimaging10050100

**Published:** 2024-04-26

**Authors:** Alireza Saberironaghi, Jing Ren

**Affiliations:** Electrical, Computer and Software Engineering, Ontario Tech University, Oshawa, ON L1G 0C5, Canada

**Keywords:** deep learning, defect detection, crack segmentation, pavement crack detection, surface defect detection, automatic defect detection, feature extraction, attention mechanism, multi-head attention

## Abstract

Detecting cracks in the pavement is a vital component of ensuring road safety. Since manual identification of these cracks can be time-consuming, an automated method is needed to speed up this process. However, creating such a system is challenging due to factors including crack variability, variations in pavement materials, and the occurrence of miscellaneous objects and anomalies on the pavement. Motivated by the latest progress in deep learning applied to computer vision, we propose an effective U-Net-shaped model named DepthCrackNet. Our model employs the Double Convolution Encoder (DCE), composed of a sequence of convolution layers, for robust feature extraction while keeping parameters optimally efficient. We have incorporated the TriInput Multi-Head Spatial Attention (TMSA) module into our model; in this module, each head operates independently, capturing various spatial relationships and boosting the extraction of rich contextual information. Furthermore, DepthCrackNet employs the Spatial Depth Enhancer (SDE) module, specifically designed to augment the feature extraction capabilities of our segmentation model. The performance of the DepthCrackNet was evaluated on two public crack datasets: Crack500 and DeepCrack. In our experimental studies, the network achieved mIoU scores of 77.0% and 83.9% with the Crack500 and DeepCrack datasets, respectively.

## 1. Introduction

Cracks in the pavement significantly signal road safety and are primarily influenced by moisture levels, the quality of construction, and traffic [1]. One study from 2006 highlighted the substantial economic impact of accidents attributed to substandard road conditions in the United States, tallying up to USD 217.5 billion [2]. Neglecting the timely maintenance of these cracks can escalate into more serious defects, compromising both traffic safety and the durability of roads, and possibly leading to accidents or the wastage of resources. As road usage increases, so do these risks, and the consequences can escalate to fatalities. Ensuring roads are in good condition is thus a crucial duty for transportation maintenance departments, and a key component of this task is crack detection. Conventional manual detection methods are plagued by several issues, such as low efficiency, disruption to regular traffic flow, time-consuming processes, and safety hazards [3]. In order to ease the load on experts and make the road inspection process more efficient, it is crucial to implement automatic crack detection.

**Figure 1:**
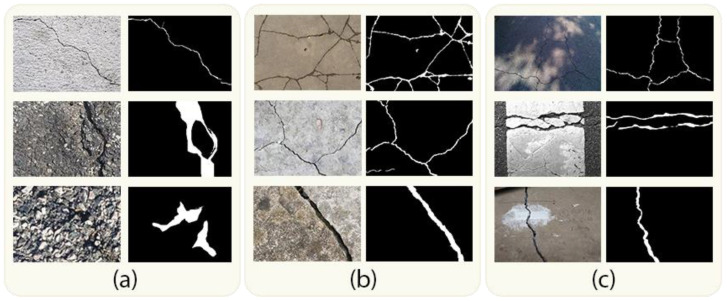
Sample images for detecting cracks in pavement surfaces: (**a**) crack variability, (**b**) varied pavement materials, (**c**) miscellaneous objects and anomalies.

As computer vision technologies have advanced, there has been a growing emphasis on leveraging these technologies for automated crack detection [4,5]. Nonetheless, the model developed for pavement crack detection is expected to face the following three principal categories of challenges, as depicted in Figure 1:

(a) Crack Variability: Cracks can come in various forms, including different lengths, widths, orientations, and curvatures, making it difficult to create a one-size-fits-all segmentation approach.
(b) Varied Pavement Materials: Different types of pavement materials, like asphalt and concrete, can exhibit varying textures and crack patterns, requiring adaptable segmentation techniques.
(c) Miscellaneous Objects and Anomalies: Pavement images can contain a variety of non-crack objects and anomalies such as road markings, potholes, paint spills, shadows, reflections, tire marks, and debris. These elements can resemble cracks in shape, size, or texture, leading to potential false positives or ambiguous detection.

Advancements in deep learning and image processing technologies have led researchers to propose a variety of automated methods for detecting pavement cracks. In the early stages of their research, Refs. [3,6] adopted threshold-based methods to detect regions of cracks. They operated under the assumption that the crack pixels were consistently darker in comparison to the surrounding areas. Various features, such as wavelet features [7], Histograms of Oriented Gradients (HOGs) [8], and Gabor filters [9], have been utilized for crack detection. While these methods are proficient in capturing the local characteristics of cracks, they tend to overlook the larger, global context of the crack. To address crack detection from a more comprehensive view, numerous studies [10,11] have incorporated both photometric and geometric attributes of pavement crack imagery into their detection algorithms. These methods strive to selectively reduce noise and enhance the continuity of the identified cracks. However, the effectiveness of these methods may diminish when applied to cracks exhibiting uneven intensity or complex topological features, leading to suboptimal detection performance. To address the previously mentioned limitations, CrackForest [12] integrates multi-level complementary features to accurately describe cracks, capitalizing on the structural information present within crack patches. This approach has demonstrated superior performance compared to other leading crack detection methods, such as Minimal Path Selection (MPS) [13], Free-Form Anisotropy (FFA) [14], CrackIT [15], and CrackTree [11]. However, CrackForest [12] relies on manually crafted features, which may lack the discriminative power needed to distinguish cracks from intricate backgrounds with subtle cues.

In newer developments, deep learning models and techniques have become widely embraced in the realm of computer vision applications. Several studies [5,16,17,18] have aimed to harness the powerful feature representation capacities of deep learning specifically for crack detection. For example, Refs. [5,17,18] employ deep learning for patch-based classification in crack detection. While this approach is effective, it can be cumbersome and susceptible to variations in the scale of the patches utilized. On the other hand, Ref. [5] approach crack detection as a segmentation task, leveraging deep learning to categorize each pixel as either part of a crack or part of the background. While this method achieves commendable results, as noted in [16], the task of crack detection presents unique challenges compared to semantic segmentation, specifically regarding the disparity between the foreground (cracks) and background elements.

In order to address the challenges of learning robust feature representations and managing the highly imbalanced classes inherent in automatic crack detection, we introduce DepthCrackNet, a model designed to automatically detect pavement cracks.

The main contributions of this paper can be outlined as follows:

- We employ the Double Convolutional Encoder (DCE) structure into our segmentation model. Comprising sequential convolution layers, this encoder is designed to facilitate enhanced feature extraction while optimizing parameter efficiency.
- We introduce the TriInput Multi-Head Spatial Attention (TMSA) mechanism, a novel attention module that processes three input feature maps simultaneously and employs multi-head attention to extract richer contextual information and enhance segmentation precision.
- We employ the Spatial Depth Enhancer (SDE) module in the encoder of our model, which skillfully extends two-dimensional feature maps into a three-dimensional context. This development aims to amplify the depth perception and spatial representation within our model.
- In empirical evaluations using publicly available crack datasets, namely Crack500 and DeepCrack, our proposed DepthCrackNet model consistently outperforms existing state-of-the-art networks in crack detection. This paper thereby presents an advancement in the realm of automated pavement crack detection.

The rest of this paper is organized as follows:

Section 2 offers a review of the relevant work in the field of pavement crack image segmentation. In Section 3, we detail the sub-modules employed in the proposed model, along with an explanation of the overall architecture. In Section 4, we present the dataset, evaluation criteria, and implementation details, as well as quantitative and visual results obtained through our experimental investigations. Moving on to Section 5, we first present the ablation analysis. Subsequently, we offer a comparative examination of our model against prior research efforts and provide a detailed analysis of situations where our model faced limitations. Finally, in Section 6, we conclude our research.

## 2. Related Studies

In this section, we begin by providing a concise overview of traditional crack detection approaches, followed by an examination of crack detection methods utilizing deep learning. The objective is to emphasize the advantages offered by deep learning techniques compared to traditional methods.

### 2.1. Traditional Crack Detection Methods

For the purposes of this work, “traditional crack detection methods” refer to approaches to detecting cracks that do not involve deep learning techniques. Over the years, a multitude of research endeavors have been channeled towards automating the detection of cracks in diverse materials and structures. The methodologies explored in these works can generally be categorized into five groups: (1) Wavelet Analysis, which leverages wavelet transforms to analyze different frequency components of an image; (2) Image Thresholding, a simple yet effective approach that segments an image based on pixel intensity; (3) Manual Feature Extraction and Classification, wherein specific characteristics are manually identified and used to train a classifier; (4) Boundary Detection Techniques, which focus on identifying and analyzing the edges within an image; and (5) Shortest-Path Techniques, which find the optimal path through an image, often used to trace cracks.

#### 2.1.1. Wavelet Analysis

Ref. [19] employed the continuous wavelet transform to automate crack detection in pavement images. However, this wavelet-based approach struggled to perform effectively on images with a wide variety of textures. In another study [7], pavement images underwent processing through a wavelet transform, breaking down the image into various frequency sub-bands. This enabled the separation of distresses and noise into wavelet coefficients of high and low amplitude, respectively.

#### 2.1.2. Image Thresholding

A series of studies [20,21,22] began with preprocessing algorithms aimed at mitigating the impact of illumination artifacts. Subsequently, thresholding was employed on each image to detect potential crack regions. These initial detections were further refined through the application of morphological operations. Ref. [23] devised a method for segmenting cracks from images that begins with the preprocessing of images using morphological filters. Following this preprocessing, dynamic thresholding was used to isolate the darker pixels, which represent potential cracks. Ref. [24] presented a methodology for segmenting crack images leveraging histograms and Ostu’s thresholding techniques. The method employed in this approach entailed partitioning the original image into four identical sub-images. Within each of these sub-images, a comprehensive analysis of cracks was conducted. Subsequently, the results from these sub-images were amalgamated to produce a conclusive predicted image. This self-guided technique demonstrated its efficiency, particularly in cases where the images exhibited a low signal-to-noise ratio, enabling approximate assessments of asphalt pavement cracks.

#### 2.1.3. Manual Feature Extraction and Classification

Most of the current crack detection methodologies depend on manually engineered features and patch-based classifiers. As illustrated in Refs. [8,25,26], hand-crafted features—such as a Histogram of Oriented Gradients (HOG) [8]—are extracted from patches of an image to serve as descriptors for cracks. Following this feature extraction, a classifier, often a Support Vector Machine (SVM), is employed to make the final determination regarding the presence of a crack.

#### 2.1.4. Boundary Detection Techniques

Ref. [27] employed the Sobel edge detection technique for crack identification post image smoothing, and utilized a bi-dimensional empirical mode decomposition algorithm for speckle noise reduction. On the other hand, Ref. [28] integrated morphological filters within their crack detection strategy, applying a modified median filter for noise eradication.

#### 2.1.5. Shortest-Path Techniques

Ref. [29] introduced a technique for identifying contour-resembling image structures via an advanced minimal-path method, reducing the need for prior information regarding the topology and endpoints of the targeted curves. Ref. [30] initiated the process by isolating potential crack regions using a windowed intensity path-based approach. Subsequently, they performed crack segmentation using a model that assessed cracks through a multivariate statistical hypothesis test. In [14], a novel approach was proposed that simultaneously considers both intensity and crack shape characteristics for crack detection. This was achieved by incorporating free-form anisotropy into the methodology.

### 2.2. Deep Learning-Based Crack Detection

In recent years, deep learning has achieved remarkable success in the field of computer vision [31]. Numerous researchers have been exploring the application of deep learning, particularly Convolutional Neural Networks, to the task of pavement crack detection. This has been motivated by the exceptional performance of these networks in computer vision tasks. Here, we present a summary of some significant contributions. Building on the rapid advancements in semantic segmentation tasks, Refs. [16,32,33] presented a crack segmentation technique inspired by SegNet [34], tailored for videos obtained from remote visual inspections. Their method pinpoints cracks by compiling the crack likelihood from several overlapping frames throughout a video sequence. Ref. [35] innovated an architectural framework that blends feature pyramid and hierarchical boosting components. In this design, the feature pyramid modules incorporate feature maps from two sequential CNN layers within the downsampling path, while the hierarchical boosting modules allocate importance weights to samples based on their level of complexity. Ref. [36] developed a data augmentation technique rooted in Generative Adversarial Networks (GANs) to address the challenge of expanding the dataset for pavement crack segmentation. This approach has proven effective in enhancing model performance. Ref. [37] formulated a semi-supervised technique for the semantic segmentation of pavement cracks. Their technique produces supervision signals for unlabeled road imagery, mitigating the constraints of manual labeling. It utilizes a fully convolutional discriminator to discern between actual ground-truth and anticipated output images. Ref. [38] created a semantic segmentation model tailored specifically for the detection of cracks in infrastructure and the precise measurement of their maximum width. Their model is built upon the synergy of a shape-sensitive kernel and a custom deep module as its foundational components. In the pursuit of automating bridge damage assessment, Ref. [39] harnessed the power of a fully Convolutional Neural Network for the comprehensive multi-class segmentation of types of bridge damage. This model holds promise as an efficient system to streamline the inspection of bridge decks. Ref. [40] collaborated the capabilities of fully Convolutional Neural Networks and multi-scale structured forests to formulate a crack segmentation model. This innovative network tackles the challenge of effectively leveraging localized information within complex backgrounds, thus surpassing the limitations inherent in traditional edge detection methods. It is important to highlight that additional refinement efforts are necessary to fortify the robustness of this classification approach. Ref. [41] introduced an automated system aimed at identifying cracks on concrete surfaces, employing the AlexNet network. They bolstered the dependability of sliding window detection by introducing an innovative probability map originating from the Softmax layer. This approach proved effective in real-world field crack detection tasks. Nonetheless, it is crucial to acknowledge that this method has constraints when it comes to classifying cracks at the pixel level, which curtails its capacity to furnish an intricate portrayal of the textural attributes of cracks. In summary, various strategies have been explored, ranging from segmentation techniques inspired by SegNet to architectural frameworks incorporating feature pyramids and hierarchical boosting. Additionally, advancements such as data augmentation using Generative Adversarial Networks (GANs) and semi-supervised approaches have been pivotal in expanding the efficacy of crack detection systems. These techniques have been tailored for specific applications, including infrastructure cracks, bridge damage assessment, and concrete surface cracks, each employing innovative components such as shape-sensitive kernels and probability maps. While they demonstrate potential, further refinement is essential to bolster these methods’ robustness and classification accuracy, especially at the pixel level.

## 3. Proposed Method

**Figure 2:**
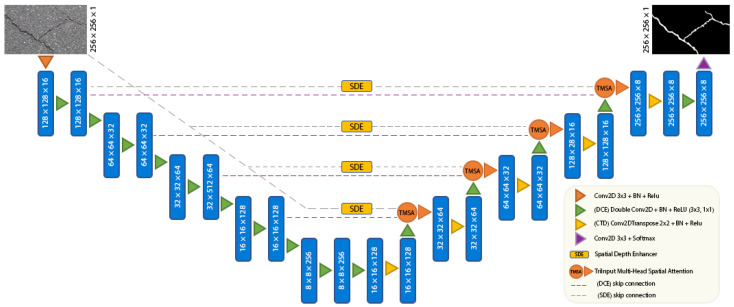
The overall architecture of the DepthCrackNet model.

In our study, we approach crack detection as a task of pixel-wise binary classification. Provided with an image that may contain a crack, our deep learning model is designed to produce a crack prediction map. In this map, regions identified with cracks are allocated higher probability scores, signaling a higher confidence that these zones contain actual cracks. On the other hand, areas without cracks are associated with lower probability scores, suggesting a reduced likelihood of crack presence. A comprehensive illustration of the architecture of our proposed model is presented in Figure 2.

As illustrated in Figure 2, the model follows a U-Net-shaped architecture. Within the encoder section, we employ a Double Convolution Encoder (DCE) module. This module integrates sequential 2D convolution, batch normalization (BN), and ReLU layers, all tailored to extract features from pavement images efficiently. To further enhance the feature extraction capability in the encoder, a Spatial Depth Enhancer (SDE) module is integrated. The 3D convolution process is applied to images through this advanced module, which enhances the feature recognition capabilities of our model. In the decoder section of our model, we introduce the TriInput Multi-Head Spatial Attention (TMSA) module, which combines feature maps. Notably, each head within this module operates independently, capturing an array of spatial relationships. The outcomes of this process are then forwarded to the Convolution Transpose Decoder (CTD), consisting of 2D transpose convolution, batch normalization (BN), and ReLU layers. The CTD network then amplifies both the width and height dimensions, leveraging these enriched features to deliver precise crack detection results. As briefly mentioned, DepthCrackNet consists of four primary components, each of which is elaborated upon in subsequent subheadings: (1) Double Convolution Encoder (DCE), (2) Spatial Depth Enhancer (SDE), (3) TriInput Multi-Head Spatial Attention (TMSA), (4) Convolution Transpose Decoder (CTD).

### 3.1. Double Convolution Encoder (DCE)

In the domain of pavement crack detection, accurately capturing the intricate details and subtle variations within cracks is crucial. Traditional Convolutional Neural Network (CNN) architectures typically employ a series of consecutive layers, including convolution, Rectified Linear Unit (ReLU) activation, and batch normalization operations, to extract features from images effectively. However, a notable challenge arises: as the network depth increases to extract more detailed semantic information, the issue of the vanishing gradient often becomes more pronounced [42,43,44]. To tackle this issue, our study presents the Double Convolution Encoder (DCE). Taking inspiration from the Inception V3 model [45], the DCE utilizes a pair of convolutional layers with diverse filter arrangements. The objective of this design is to adeptly isolate spatial details while avoiding a significant escalation in parameter count. Moreover, this configuration empowers the Double Convolution Encoder (DCE) to circumvent the challenges frequently encountered in deep CNNs, thereby demonstrating excellence in feature identification even in scenarios where the volume of training data is limited [46]. Table 1 shows the layers and filters of the DCE module used in DepthCrackNet. At their core, Convolutional Neural Networks (CNNs) are architectural structures primarily composed of three essential components: (1) convolutional layers, (2) batch normalization, and (3) activation functions [47].

#### 3.1.1. Convolution Layer

**Figure 3:**
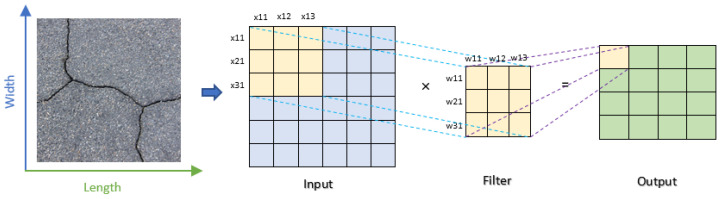
Illustration of the 2D convolution process in a convolutional layer.

The convolutional layer is the central element of a CNN, acting by applying a convolution operation to the input data, which effectively serves as a specific type of filtering process. This operation is visually depicted in Figure 3. Throughout the training phase, the model fine-tunes the filter weights, empowering them to identify and emphasize the features most relevant to the problem at hand. In this scenario, “w” symbolizes the weight, “x” designates the input data, “b” represents the bias value, and N _Output_ is the resulting output, as referenced in Equation (1).

N _Output_ = w × x + b (1)

#### 3.1.2. Batch Normalization Layer

Batch normalization plays a crucial role in enhancing the stability and efficiency of Neural Networks. This step involves normalizing the outputs from preceding layers, which helps to ensure a consistent mean and variance for the inputs to each subsequent layer. This normalization process is instrumental in alleviating the internal covariate shift, which in turn accelerates the training process and decreases the dependence on initial weight configurations. Additionally, batch normalization frequently results in smoother loss function landscapes, thereby simplifying the optimization process.

#### 3.1.3. Activation Layer

After the convolutional operation, the data are forwarded through the activation layer. In this step, a designated transformation is applied to the output data from the prior layer, which infuses non-linearity into the model’s calculations. A common activation function used for this purpose is the Rectified Linear Activation Unit (ReLU), which effectively nullifies negative values by setting them to zero. Although ReLU is extensively employed, alternative functions such as “tanh” and “sigmoid” exist, and these serve comparable roles by constraining the input data within a specified range.

### 3.2. Spatial Depth Enhancer (SDE)

**Figure 4:**
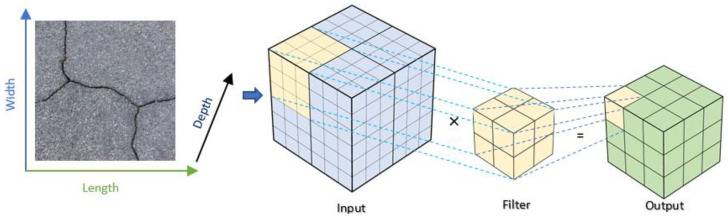
Illustration of a 3D convolution operation.

To enhance feature extraction and accentuate depth-related attributes in pavement crack images, we integrated the Spatial Depth Enhancer (SDE) into the encoder section of our segmentation model. The SDE’s fundamental goal is to deepen the spatial perspective of features in an efficient computational manner. This is achieved by transitioning images from traditional 2D convolutions to 3D convolutions. To achieve this, the input image is partitioned into N × N segments, which are then introduced as a third dimension. The outcome of this process closely resembles 3D biomedical images [48]. Upon receiving an input tensor, the SDE module first adjusts its dimensions to be conducive to 3D convolution operations. This modification prepares the tensor for depth-wise transformations in subsequent steps. Following this reshaping, a 3D convolution operation is initiated. By introducing an additional depth dimension, unlike conventional 2D convolution methods, this approach extracts spectral and spatial features from disparate regions in the image while maintaining the integrity of the original image structure. Figure 4 shows an example of a 3D convolution operation. Notably, the depth of the convolution filter is dynamically derived from the input’s channels, promoting adaptability and computational efficiency. Finally, the tensor, post its 3D convolution and activation, is reshaped back to a 2D format. This ensures compatibility and seamless integration with subsequent layers in the model (TMSA module). Table 2 presents the layers and filters of the Spatial Depth Enhancer (SDE) module utilized in the model. In essence, the SDE module empowers our segmentation model with a more comprehensive spatial understanding of the input image. This proves invaluable in identifying subtle and intricate crack patterns on pavements that might be elusive to models reliant solely on 2D convolution layers.

### 3.3. TriInput Multi-Head Spatial Attention (TMSA)

**Figure 5:**
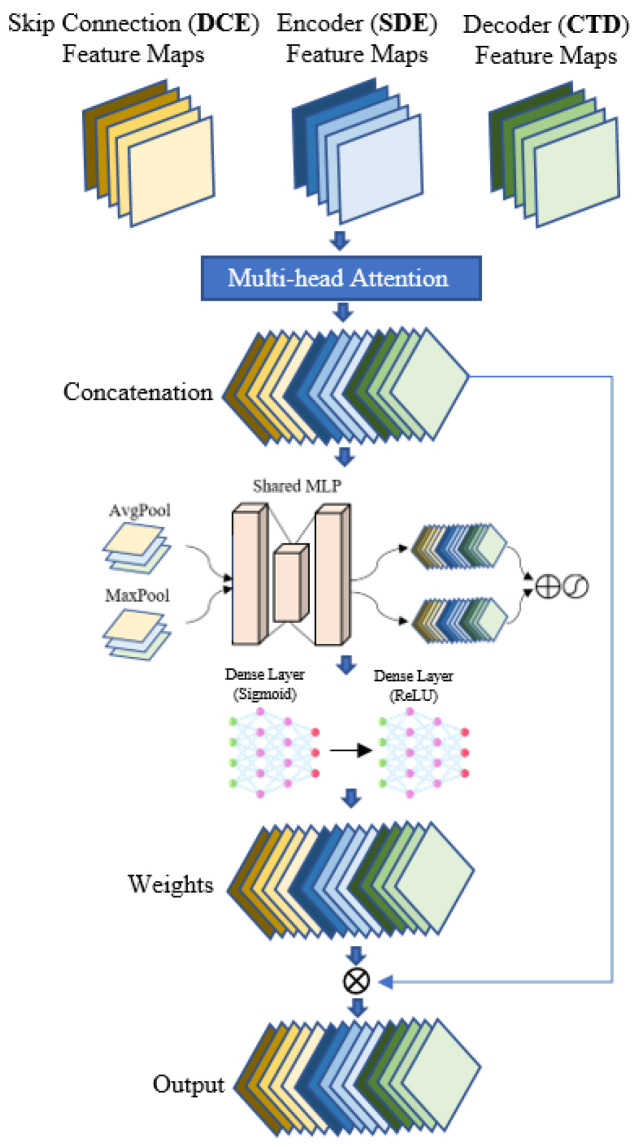
Overall structure of the TriInput Multi-Head Spatial Attention (TMSA) module.

Attention mechanisms have revolutionized the way deep learning models interpret and prioritize information [31,49]. Instead of treating all inputs uniformly, attention allows a model to focus selectively on specific segments that are most pertinent for a given task. A multi-head attention mechanism takes this concept a step further. Rather than having a singular focus or perspective, multi-head attention enables the model to possess multiple “points of view” [50,51]. This ensures that the resulting feature map captures a rich set of spatial information from various perspectives. In the realm of crack pavement detection through segmentation models, the TriInput Multi-Head Spatial Attention (TMSA) module emerges as a pivotal innovation tailored for the decoder section. Details such as color, edge, and texture are encapsulated within spatial information, whereas semantic details contain contextual information crucial for classification tasks, albeit often lacking precision in terms of location and shape [52]. Conversely, spectral features capture the spatial connections among different points within the input image. This is achieved by means of reshaping and 3D convolution processes, as discussed in Ref. [53]. Since these features are derived through distinct methods, they inherently contain diverse content. In this study, rather than merging these feature maps directly, a unique approach was employed by forming a 3D-input multi-head attention structure. This TMSA module is fundamentally constructed upon the principles of the squeeze-and-excitation framework [54], rather than directly merging the diverse feature maps. This module efficiently concatenates three different feature maps. Initially, the feature maps extracted from the double Conv2D layers (DCE) are propagated through the skip connections in the encoder. Subsequently, the spatial feature maps, produced by the Spatial Depth Enhancer (SDE) module, highlight depth-related intricacies. Lastly, the feature maps generated from the Conv2DTranspose (CTD) module within the decoder are intended to recover spatial details that may have been attenuated during encoding. The core idea behind the TMSA module is to employ multi-head attention, a mechanism enabling the model to focus on several spatial positions simultaneously. In the TMSA module, each head starts by sequentially concatenating all three inputs, resulting in a unified feature map. Subsequently, both 2D Global Average Pooling and Max Pooling layers are applied to calculate the average and maximum values across spatial dimensions within this unified feature map. This step generates a comprehensive representation of the merged feature maps, which is then channeled through two consecutive dense layers, with a ReLU activation function inserted in between. The output from these dense layers is subjected to a sigmoid activation, yielding weights corresponding to the spatial locations within the unified feature map. These weights are then reshaped and broadened to align with the spatial dimensions of the initial input feature map. The final step involves an element-wise multiplication between the expanded weights and the unified feature map. This ensures that each spatial location within the map is assigned a weight reflecting its relative importance. After all of the heads have completed their processing, their individual outputs are aggregated to construct the final attention-enhanced feature map. Through its intricate attention mechanism, the TMSA module guarantees that the resulting feature map emphasizes important regions, while also harmoniously integrating various spatial and depth-related cues. This amalgamation of spatial information is designed to significantly bolster the segmentation model’s proficiency in the precise detection of cracks in pavements. Figure 5 demonstrates the overall structure of the TriInput Multi-Head Spatial Attention (TMSA) module.

### 3.4. Convolution Transpose Decoder (CTD)

The architecture of DepthCrackNet heavily relies on the DCE and SDE modules within the encoder section, as they play a crucial role in generating robust feature maps. Situated in the decoder section of the DepthCrackNet model, the Convolution Transpose Base Decoder (CTD) network capitalizes on these features to yield results in crack detection. The CTD module is carefully constructed to up-scale and refine the feature maps in the decoding section of our architecture. It consists of a series of operations: transpose convolution, batch normalization, and the application of Rectified Linear Unit (ReLU) activation. These operations are aimed at restoring spatial details that might have been lost during the encoding phase. At the core of the CTD module is the Conv2DTranspose layer. This layer uses transposed convolutions, also known as deconvolutions, to expand the spatial dimensions of the feature maps. To achieve this up-scaling, a kernel of a (4,4) size and a stride of (2,2) are employed, effectively doubling the spatial resolutions. After batch normalization, ReLU activation is applied, introducing non-linearity into the feature maps and enabling the capture of complex patterns and relationships within the data. An important feature of the CTD module is its seamless integration with preceding structures in the model, particularly the TriInput Multi-Head Spatial Attention (TMSA) and the Double Convolutional Encoder (DCE) components. This integration, achieved through the strategic use of TMSA, allows the module to skillfully merge the upsampled tensor with the provided skip connections. Consequently, the decoder harnesses high-level features from deeper layers and synergizes them with spatial details from earlier stages of the network, a process crucial for retaining the level of detail necessary for accurate crack detection. The CTD network is organized into five levels. Table 3 presents the layers and filters of the Convolution Transpose Decoder (CTD) module utilized in our model. In the CTD network, the initial four levels encompass layers of convolution transpose, TMSA, and DSC. As each stage commences, the feature map undergoes a series of transformations. Conv2DTranspose layers are employed to expand both its width and height. The TMSA layer plays a crucial role in amalgamating the emerging high-level feature map with the low-level feature maps, as well as the spectral and spatial feature maps. Upon reaching the final level of the CTD network, referred to as the output level, the feature map undergoes additional processing. This involves Conv2DTranspose, Batch Normalization (BN), and Rectified Linear Unit (ReLU) operations to prepare it for crack detection. Subsequently, the feature map is further refined by passing it through the DSC layer. The resulting feature map, which matches the dimensions of the input image, comprises 16 channels. During the phase of pixel-wise classification, a 1 × 1 convolution (Conv) operation is applied to the feature map, followed by the application of a Softmax function. This series of operations culminates in the creation of a 256 × 256 × 2 output matrix.

## 4. Experimental Results

A performance assessment of the DepthCrackNet model was carried out by comparing its performance on two publicly accessible datasets, Crack500 [35] and DeepCrack [55], against the performance of R2U-Net [56], Attention U-Net [57], TransUNet [58], and Swin-Unet [59], which are commonly used in the literature. In this study, Section 4.1 outlines the specifics of the datasets used, while Section 4.2 describes the evaluation metrics employed. The implementation particulars and the training process are elaborated in Section 4.3. Finally, Section 4.4 is dedicated to presenting and analyzing the results of the conducted experiments.

### 4.1. Datasets

**Figure 6:**
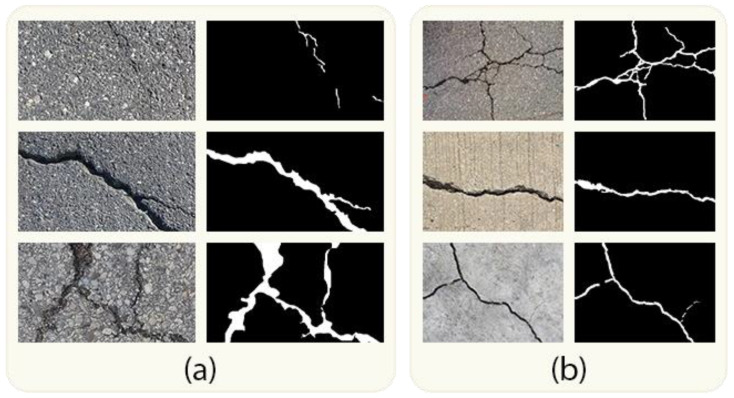
Example images and corresponding ground-truth data from the datasets employed in this study: (**a**) includes images from the Crack500 dataset, while (**b**) contains images from the DeepCrack dataset.

We conducted experiments to evaluate our proposed method using two publicly available pavement crack datasets: Crack500 [35] and DeepCrack [55]. We divided these datasets into three distinct sets: a training set, a validation set, and a test set, following a 6:2:2 ratio [60]. Table 4 provides a summary of these two datasets, while Figure 6 displays sample images and corresponding ground truths from the datasets employed in this study.

#### 4.1.1. Crack500

The Crack500 dataset [35] comprises a total of 500 images, each possessing a resolution close to 2000 × 1500 pixels. These images were captured within the confines of the Temple University premises using a mobile phone. In order to accommodate computing resource limitations, each image was divided into 16 distinct, non-overlapping sections. Only the sections that contained more than 1000 crack pixels were retained. Importantly, pixel-level annotations were meticulously added to every crack image. As a result, the Crack500 dataset currently comprises a total of 3368 crack images.

#### 4.1.2. DeepCrack

Comprising 537 crack images, the DeepCrack dataset [55] features complex backgrounds and a range of crack scales, offering a more comprehensive representation of crack characteristics. This dataset includes three textures—bare, dirty, and rough—as well as two types of scenes, namely concrete and asphalt. The cracks within the images vary in width, ranging from a single pixel up to 180 pixels. In each image, the cracked area represents only a small percentage of the total area, mirroring real-world conditions. Each of these crack images has been meticulously annotated by hand, resulting in binary image representations.

### 4.2. Evaluation Metrics

To evaluate the performance of our segmentation model, we depended on specific metrics. These included Precision, Recall, F1 score, and mIoU. Precision is used to determine the efficiency with which defects are classified, while Recall is employed to gauge the effectiveness of identifying negative samples. The F1 score serves as a harmonizing metric between Precision and Recall, providing a measure that assesses the model’s ability to accurately and reliably differentiate between the segmented regions and the true target regions within the image. Mean Intersection over Union (mIoU), conversely, is used to assess the extent of overlap between the model’s predicted segmentation and the actual ground truth, serving as an indicator of the model’s spatial accuracy in delineating objects or defects.

### 4.3. Implementation Details and Training

In this section of our research, we provide detailed insights into the specific configuration of our model, including the hyperparameters that were chosen for the training process. Our proposed model underwent experimental testing using the TensorFlow framework. This testing was conducted on a computing setup equipped with an NVIDIA 80 GB GPU card and 90 GB RAM, which operated within the Paperspace platform environment. For the training phase of the proposed networks, we set the batch size and the number of epochs to 32 and 200, respectively. We employed the Adam optimization algorithm to fine-tune the network parameters. In our model, we set the number of heads for the TriInput Multi-Head Spatial Attention (TMSA) to four. Following a comprehensive set of ablation experiments, which involved various loss functions, we ultimately opted for a weighted hybrid loss function. During the training of our segmentation model, this loss function demonstrated effectiveness in balancing the learning process by considering both pixel-wise classification accuracy and the spatial coherence between the predicted and actual ground-truth segmentations. Specifically, this loss function was defined as (0.9 Binary Cross-Entropy Loss + 0.1 × Dice Loss). Here, the Binary Cross-Entropy Loss focuses on the accuracy of individual pixel classifications, while the Dice Loss is designed to enhance the resemblance between the predicted segmentation regions and the ground truth, thus promoting more cohesive segmentation results. To fine-tune the learning rate and determine the optimal number of epochs for training, we incorporated the ReduceLROnPlateau and EarlyStopping callback functions. The ReduceLROnPlateau function adjusts the learning rate by multiplying it by a specific factor when there has not been a decrease in the loss value for a predetermined “patience” number of epochs. Similarly, the EarlyStopping function halts the training process when appropriate. For this study, we set the factor and patience values for the ReduceLROnPlateau function to 0.5.

### 4.4. Results

In this section, we present the results obtained from utilizing the Crack500 and DeepCrack datasets, showcasing visual and numerical outcomes in Section 4.4.1 and Section 4.4.2, respectively.

#### 4.4.1. Crack500

**Figure 7:**
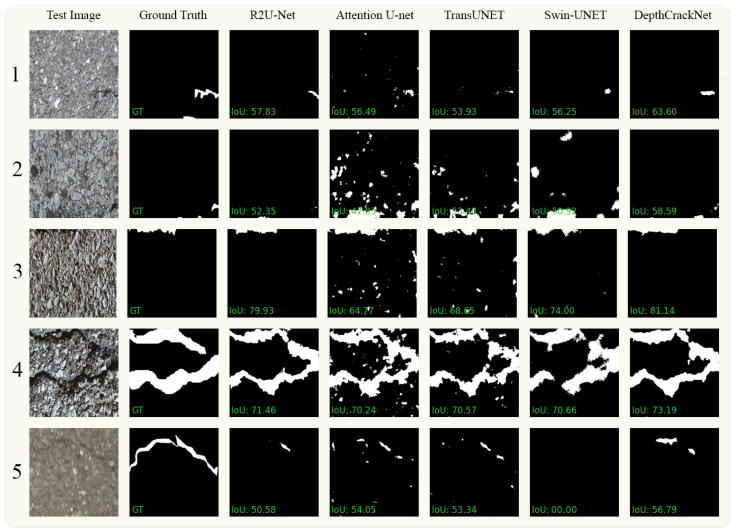
Visual comparison between the DepthCrackNet model and several leading models using the Crack500 dataset.

In Figure 7, we present a visual comparison of the ground-truth data extracted from the Crack500 dataset alongside segmentation results obtained from various methods, including our proposed DepthCrackNet. The figure is organized as follows: The first and second columns display the original images and their corresponding ground-truth segmentations. Columns 3 to 6 showcase the results achieved using the R2U-Net, Attention U-Net, TransUNet, and Swin-Unet methods, respectively. Finally, in column 7, we present the segmentation outcomes produced by our novel DepthCrackNet. This visual representation highlights the diverse and complex challenges encountered in pavement crack detection within the Crack500 dataset. In the first row, entailing small crack detection, our model outperforms with an IoU of 63%, demonstrating a more profound discernment of fine-grain features compared to the others, where R2U-Net, Attention U-Net, TransUNet, and Swin-Unet register IoUs of 57%, 56%, 53%, and 56%, respectively. This advantage is sustained in the second row, which introduces background similarity issues, with our model attaining an IoU of 58%, marginally superior to Swin-Unet at 56%, showcasing the model’s resilience to background noise. Significantly, in the third row, which encompasses tiny cracks on textured pavement, our model excels with an IoU of 81%, indicating its stronger ability to identify and delineate subtle defects amidst textured backgrounds. This is a noteworthy performance, as even the promising R2U-Net lags behind at 79%. In the segmentation of thick cracks, shown in the fourth row, our model’s IoU of 73.19% asserts its robustness in capturing prominent defect features against the competitive architectures, which hover around the 70% mark. The fifth row highlights the challenge posed by different pavement materials, wherein our model performs better with an IoU of 56%, while Swin-Unet fails notably with an IoU of 0. This underscores the need for a versatile architecture to handle various material textures in pavement crack detection.

Table 5 demonstrates a comparative analysis of our proposed model with existing state-of-the-art models on the Crack500 dataset. The results encapsulate several performance metrics, among which the mean Intersection over Union (mIoU) emerges as a significant indicator of the models’ competence in delineating the crack regions accurately. Our model conspicuously outshines the others with an mIoU of 0.77, indicating a superior balance in accurately identifying both crack and non-crack regions. R2U-Net follows closely with an mIoU of 0.7345, yet lags behind by a discernible margin. TransUNet also shows competitive performance with an mIoU of 0.6908, yet its score is markedly overshadowed by the higher mIoU attained using our model, underscoring the efficacy of the architectural enhancements we incorporated. Attention U-Net, despite its focus mechanism, manifests a lower mIoU of 0.6558, suggesting potential room for improvement in handling the complexities inherent in the Crack500 dataset. On the other hand, Swin Transformer registers an mIoU of 0.6638, which, while being respectable, hints at challenges in adapting transformer architectures for this specific task. The superior mIoU score of our model is a testament to its robustness and adeptness in handling the challenging scenarios of pavement crack detection. It reflects a well-rounded performance across Precision, Recall, and F1 score as well, with a notable Precision of 0.87, significantly higher than the Precision scores of all other compared models.

#### 4.4.2. DeepCrack

**Figure 8:**
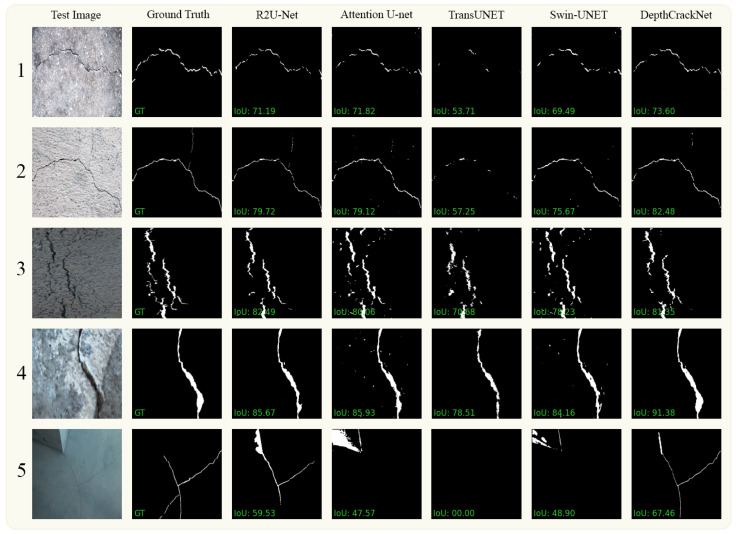
Visual comparison between the DepthCrackNet model and several leading models using the DeepCrack dataset.

Figure 8 showcases an evaluation of our proposed segmentation model on the challenging DeepCrack dataset against contemporary state-of-the-art models including R2U-Net, Attention U-Net, TransUNet, and Swin-Unet. In the first row, dedicated to a background similarity challenge, our model registers an IoU of 73%, superseding all other compared models, with Attention U-Net trailing closely behind at 71.82%. This clearly underscores our model’s superior capability in discriminating crack defects amidst background noise, a crucial facet for real-world deployment. The second row, highlighting thin cracks, again sees our model leading with an impressive IoU of 82.48%, substantiating its adeptness at detecting fine-grained defect features. This stands in stark contrast to TransUNet, which lags behind at 57.25%, suggesting potential shortcomings in capturing minor defects. In the third row, featuring diverse crack sizes, a common occurrence in pavement crack detection, our model maintains its high performance with an IoU of 81%, albeit slightly lower than R2U-Net at 82%. However, it significantly surpasses TransUNet, which falters with an IoU of 70%, hinting at our model’s better adaptation to size variability. Proceeding to the fourth row, evaluating thick cracks, our model manifests a commanding lead with an IoU of 91.38%. The closest contender, Attention U-Net, trails considerably behind at 85.93%, reaffirming our model’s robustness in delineating pronounced defects. Lastly, the fifth row accentuates the challenge of very thin cracks. Despite the difficulty, our model markedly outperforms with an IoU of 67.46%, while TransUNet disastrously fails to catch any cracks with an IoU of 0. This stark difference underscores the architectural fortitude of our model in confronting one of the most challenging defect types in pavement crack detection.

Table 6 gives the numerical results of the DeepCrack dataset experiment. Our model shows an mIoU of 0.839, performing better compared to other models in effectively delineating crack regions from the background. The R2U-Net model trails closely behind with an mIoU of 0.7923, albeit with a margin that still underlines the superior detection capabilities of our model. Notably, our model demonstrates a balanced performance with a Precision of 0.819 and Recall of 0.849, indicating its adept handling of both false positives and false negatives, which is further corroborated by its impressive F1 score of 0.833. Attention U-Net, known for its attention mechanism to refine feature representations, secures an mIoU of 0.7579. Despite its focus-driven architectural strength, it falls short in comparison to our model, particularly in handling the intricate crack patterns inherent in the DeepCrack dataset. TransUNet follows closely with an mIoU of 0.7503, displaying a decent capability in identifying crack regions. Interestingly, Swin Transformer, with its inherent capacity for long-range interactions, attains a lower mIoU of 0.6901. Its suboptimal Recall of 0.5448 distinctly highlights the challenges faced by transformer-based architectures in accurately detecting all crack instances, especially amidst varied pavement textures and crack sizes.

In comparison to several prominent neural network architectures for image segmentation, the proposed DepthCrackNet model demonstrates a notably efficient parameter utilization. While Attention U-Net and TransUNet employ 31.9 million and 434.1 million parameters, respectively, our model significantly reduces the parameter count to 5.6 million. Moreover, compared to R2U-Net with 23 million parameters and Swin-Unet with 9.3 million parameters, our DepthCrackNet model maintains a competitive advantage with its streamlined parameter configuration. This parsimonious parameterization not only minimizes the computational overhead but also alleviates the burden of data requirements for training.

## 5. Discussion and Analysis

This section is organized into three subsections to provide a comprehensive analysis. Section 5.1 delves into ablation studies to understand the contribution of each component of the model. Section 5.2 focuses on reviewing prior studies associated with the datasets. Subsequently, Section 5.3 examines scenarios where the proposed model either falls short or inaccurately identifies cracks.

### 5.1. Ablation Analysis

The challenge of automatically detecting pavement cracks is both crucial and complex, and to address this, the DepthCrackNet model introduces three key modules. To discern the impact and importance of these modules, a thorough ablation analysis was undertaken, focusing on the Double Convolution Encoder (DCE), the TriInput Multi-Head Spatial Attention (TMSA) module, and the Spatial Depth Enhancer (SDE) module. The results of these ablation experiments are summarized in Table 7. This process involved evaluating the model’s performance based on the addition of each specific component. In the case of DepthCrackNet, this meant measuring the outcomes on the Crack500 and DeepCrack datasets, both of which are benchmark datasets in the realm of crack detection. Starting with the Baseline Convolution Encoder, which represents the initial configuration without any additional modules, the results provided a benchmark for comparison. This baseline setup, although effective to some extent, lacked the advanced mechanisms needed for precise feature extraction and spatial attention. Upon integrating the Double Convolution Encoder (DCE), a notable improvement in performance metrics such as Precision, Recall, and F1 score was observed. This module facilitated a more efficient extraction of features from the pavement images, leading to better crack detection. The subsequent addition of the TriInput Multi-Head Spatial Attention (TMSA) module further elevated the model’s capability to capture intricate spatial relationships within the feature maps. As evidenced by the results, this augmentation significantly boosted both Precision and Recall, indicating a more comprehensive understanding of crack patterns. Furthermore, the inclusion of the Spatial Depth Enhancer (SDE) module marked another leap in performance. By leveraging 3D convolution processes, this module enhanced the model’s ability to recognize nuanced features within the pavement images, resulting in a substantial increase in mIoU. The combined effect of all three modules yielded the most remarkable results, with Precision, Recall, F1 score, and mIoU reaching their peak values.

In conclusion, this ablation study unequivocally validates the importance of each module introduced in DepthCrackNet. The DCE, TMSA, and SDE significantly and cumulatively contribute to the model’s overall robustness and accuracy. It would be intriguing for future studies to further optimize these components or introduce novel modules to enhance crack detection precision.

### 5.2. Evaluating the Proposed Model against Previous Studies

Detecting cracks in pavements is a crucial aspect of quality control. Numerous approaches leveraging image processing and machine learning methodologies have been created to streamline and automate the detection process. In this research, a model named DepthCrackNet was designed specifically for automatic surface defect identification. This study carried out extensive experimental evaluations of the proposed model using two widely recognized public datasets in the literature: Crack500 and DeepCrack. Table 8 displays a comparison of results from prior studies employing these datasets, offering additional insights into the assessment of the proposed model’s performance.

As seen from Table 8, on the Crack500 dataset, the proposed DepthCrackNet model achieved a Precision of 0.870, significantly outperforming all other models listed, with the next highest being the 0.830 score achieved using the model presented in [62] that employs a feature pyramid network with a self-guided attention refinement module. However, our model falls short in Recall (0.641) compared to some models, most notably the model in [63], which achieved a Recall of 0.800 using DeepLab with Multi-Scale Attention. The F1 score of DepthCrackNet (0.738) is competitive but does not reach the F1 score of 0.794 achieved by the model in [62]. The mean Intersection over Union (mIoU) of our model (0.770) is the highest among all models, showcasing a notable improvement over the existing methods. The previous highest mIoU was 0.653 achieved by the model in [65]. Transitioning to the DeepCrack dataset, the proposed model achieved a commendable Precision of 0.819, although it is superseded by the model in [68] with a Precision of 0.883 employing an Attention Module and Focal Loss Function. On the DeepCrack dataset, our model exceled in Recall with a score of 0.849, which is the highest among all models, indicating its superior capability in identifying a higher number of actual positive crack instances. The F1 score of our model (0.833) is quite competitive, only being surpassed by the model in [70], which employs a Morphology Branch and Shallow Detail Branch, with an F1 score of 0.875. The mIoU score of DepthCrackNet (0.839) surpasses all other models listed, indicating a superior overlap between the predicted segmentation and the ground truth. The comparative analysis results reveal that the integration of 3D spatial features and a multi-head attention mechanism in our model significantly contributes to its effectiveness in identifying pavement cracks accurately. Despite the lower Recall value on the Crack500 dataset, the higher Precision and mIoU scores underline the model’s ability to provide more accurate segmentation, reducing the false positives, which is crucial for real-world deployment in pavement maintenance systems.

### 5.3. Analysis of Failures

**Figure 9:**
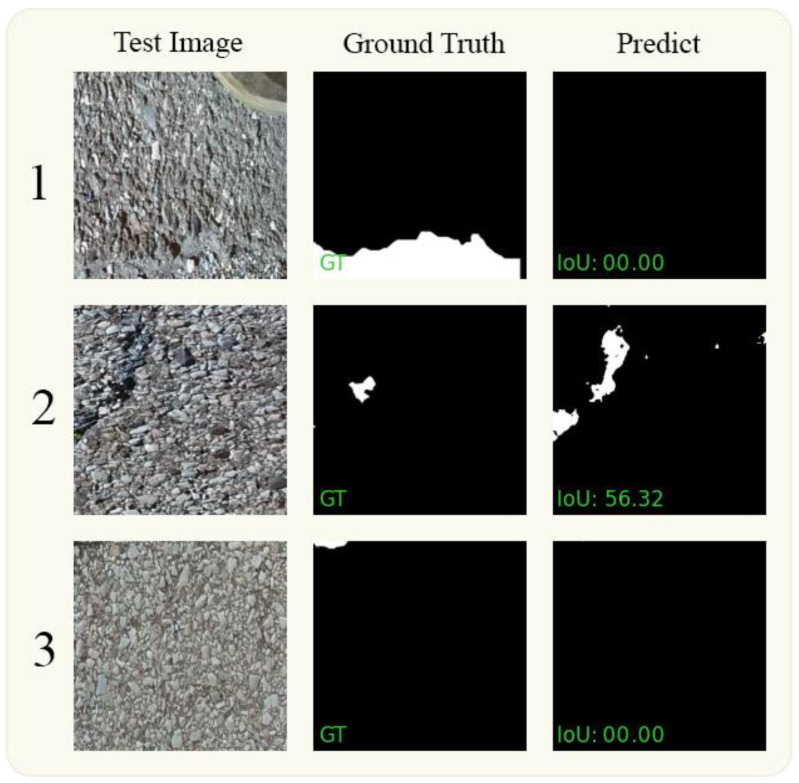
The visual outcomes of the failures are depicted in rows 1 and 2 for the Crack500 dataset and in row 3 for the DeepCrack dataset.

Despite the notable success of our proposed model in detecting pavement cracks across different datasets, it is imperative to analyze the instances where the model failed to deliver as expected to identify areas of improvement. In Figure 9, we illustrate three samples where our model struggled to accurately detect cracks. In the first row of Figure 9, our model could not identify any cracks, highlighting a limitation in its ability to discern subtle features within challenging images. This failure could be attributed to certain challenging features in the images that may have confused the model, such as similar background textures or low contrast between the cracks and the surrounding area, revealing a constraint in the model’s adaptability to distinguish visual contexts. Understanding the specifics of this failure is crucial for improving the model’s robustness in future iterations, underscoring the necessity for enhanced feature extraction mechanisms to handle such complexities effectively. The second row of Figure 9 shows a scenario where our model managed to achieve an Intersection over Union (IoU) of 56.32, indicating moderate success in crack detection. While this demonstrates some capability of the model to detect cracks, the IoU score reveals a significant room for improvement, indicating a limitation in the model’s Precision and Recall balance. The third row of Figure 9 again depicts a case where the model could not detect any cracks, highlighting a persistent challenge in detecting certain types of cracks or visual conditions within the image and signifying a limitation in the model’s generalization ability. Identifying the underlying causes of these failures will be pivotal in refining the model for better accuracy and reliability, emphasizing the need for targeted enhancements in model architecture and training strategies. These failure cases provide valuable insights into the limitations of our current model, emphasizing the necessity for comprehensive solutions to address the identified constraints. Future work should delve into addressing these issues, possibly through augmenting the training data with more varied examples of cracks to enhance the model’s adaptability, refining feature extraction mechanisms to improve its sensitivity to subtle cues, and exploring advanced postprocessing techniques to mitigate false negatives and bolster its overall performance in crack detection.

## 6. Conclusions

This work introduced DepthCrackNet, a novel U-Net-shaped model, aimed at automating the essential task of pavement crack detection to enhance road safety. The model’s architecture, comprising a Double Convolution Encoder (DCE), TriInput Multi-Head Spatial Attention (TMSA) module, and Spatial Depth Enhancer (SDE) module, is designed to navigate the challenges posed by crack variability and miscellaneous on-road anomalies. DepthCrackNet was rigorously evaluated on two public datasets, Crack500 and DeepCrack, achieving promising mIoU scores of 77.0% and 83.9%, respectively. These outcomes, along with the results of a comparative analysis with existing models, underscore DepthCrackNet’s potential for real-world deployment in pavement maintenance systems. The results advocate for further research to optimize DepthCrackNet for real-time applications and explore its performance across diverse pavement types and conditions. Through DepthCrackNet, a significant step towards automated, accurate, and efficient pavement crack detection has been made, aligning with the broader goal of ensuring road safety.

## Figures and Tables

**Table 1:** Layers of the Double Convolution Encoder (DCE) module used in the DepthCrackNet model.

Level	Layer	Filter	Output Dimensions
Input	-	-	256 × 256 × 1
Level 1	Conv 2D 3 × 3 + BN + ReLU	16	128 × 128 × 16
	DCE	16	128 × 128 × 16
Level 2	DCE	32	64 × 64 × 32
	DCE	32	64 × 64 × 32
Level 3	DCE	64	32 × 32 × 64
	DCE	64	32 × 32 × 64
Level 4	DCE	128	16 × 16 × 128
	DCE	128	16 × 16 × 128
Level 5	DCE	256	8 × 8 × 256
	DCE	256	8 × 8 × 256

**Table 2:** Layers of the Spatial Depth Enhancer (SDE) module used in the DepthCrackNet model.

Level	Layer	Filter	Output Dimensions
Level 1	SDE	8	128 × 128 × 16
Level 2	SDE	4	64 × 64 × 32
Level 3	SDE	2	32 × 32 × 64
Level 4	SDE	1	16 × 16 × 128

**Table 3:** Layers of the Convolution Transpose Decoder (CTD) module used in the DepthCrackNet model.

Level	Layer	Filter	Output Dimensions
Level 1	Conv2DTranspose + BN + ReLU	128	16 × 16 × 128
	TMSA	-	16 × 16 × 128
Level 2	Conv2DTranspose + BN + ReLU	64	32 × 32 × 64
	TMSA	-	32 × 32 × 64
Level 3	Conv2DTranspose + BN + ReLU	32	64 × 64 × 32
	TMSA	-	64 × 64 × 32
Level 4	Conv2DTranspose + BN + ReLU	16	128 × 128 × 16
	TMSA	-	128 × 128 × 16
Level 5	Conv2DTranspose + BN + ReLU	8	256 × 256 × 8
	Conv2D + Softmax	1	256 × 256 × 2

**Table 4:** Overview of the crack datasets utilized in our experiments.

Dataset	Resolution	Images	Training	Validation	Test
Crack500 [35]	640 × 360	3368	2020	647	647
DeepCrack [55]	Variable	537	429	54	54

**Table 5:** Evaluation results for the DepthCrackNet model alongside other models on the Crack500 dataset.

Model	Precision	Recall	F1	mIoU
Attention U-Net [57]	0.4998	0.7294	0.5931	0.6558
R2U-Net [56]	0.8121	0.6710	0.7349	0.7345
TransUNet [58]	0.6710	0.6417	0.6561	0.6908
Swin-Unet [59]	0.6701	0.5767	0.6199	0.6638
DepthCrackNet	0.8703	0.6411	0.7383	0.7700

**Table 6:** Evaluation results for the DepthCrackNet model alongside other models on the DeepCrack dataset.

Model	Precision	Recall	F1	mIoU
Attention U-Net [57]	0.8180	0.8134	0.8157	0.7579
R2U-Net [56]	0.8795	0.8909	0.8852	0.7923
TransUNet [58]	0.8231	0.7668	0.7940	0.7503
Swin-Unet [59]	0.8194	0.5448	0.6545	0.6901
DepthCrackNet	0.8193	0.8491	0.8339	0.8393

**Table 7:** Ablation experimental results using the Crack500 and DeepCrack datasets.

Methods	Dataset	Precision	Recall	F1	mIoU
Baseline Convolution Encoder + CTD	Crack500	0.845	0.721	0.779	0.719
DCE + CTD		0.855	0.735	0.790	0.728
DCE + CTD + TMSA		0.867	0.753	0.805	0.745
DCE + CTD + TMSA + SDE		0.870	0.641	0.738	0.770
Baseline Convolution Encoder + CTD	DeepCrack	0.812	0.743	0.776	0.782
DCE + CTD		0.825	0.758	0.790	0.785
DCE + CTD + TMSA		0.838	0.772	0.803	0.802
DCE + CTD + TMSA + SDE		0.819	0.849	0.833	0.839

**Table 8:** Comparative results from previous studies utilizing the Crack500 and DeepCrack datasets.

References	Methods	Dataset	Precision	Recall	F1	mIoU
[61]	CNN, Pyramid Attention Network	Crack500	0.816	0.765	-	0.6235
[62]	Feature pyramid network, self-guided attention refinement module		0.830	0.796	0.794	-
[63]	DeepLab with Multi-Scale Attention		0.695	0.800	0.744	0.559
[64]	Unet-based method		-	-	-	0.60
[65]	CNN model		0.807	0.773	-	0.653
[66]	Self-Attention-based Efficient U-Net		-	-	0.775	0.663
[67]	ECA Channel Attention Module and FCNhead Decoding Dock		-	-	-	0.6397
[68]	RUC-Net with scSE Attention Module		0.698	0.761	0.729	0.573
[69]	Joint Topology-preserving and Feature-refinement Network		0.68.81	0.690	0.657	-
Proposed DepthCrackNet	3D Spatial Features and Multi-Head Attention Mechanism		0.870	0.641	0.738	0.770
[68]	Attention Module and Focal Loss Function	DeepCrack	0.883	0.812	0.846	0.733
[70]	Morphology Branch and Shallow Detail Branch		-	-	0.875	0.779
Proposed DepthCrackNet	3D Spatial Features and Multi-Head Attention Mechanism		0.819	0.849	0.833	0.839

## Data Availability

The datasets utilized in this study are publicly available and accessible at the following links: DeepCrack: https://github.com/yhlleo/DeepCrack (accessed on 23 April 2024); Crack500: https://github.com/fyangneil/pavement-crack-detection (accessed on 23 April 2024).
